# Bioinformatics as a driver, not a passenger, of translational biomedical research: Perspectives from the 6^th ^Benelux bioinformatics conference

**DOI:** 10.1186/2043-9113-2-7

**Published:** 2012-03-13

**Authors:** Francisco J Azuaje, Michaël Heymann, Anne-Marie Ternes, Anke Wienecke-Baldacchino, Daniel Struck, Danièle Moes, Reinhard Schneider

**Affiliations:** 1 Department of Cardiovascular Diseases, Public Research Centre for Health (CRP-Santé), 1150 Luxembourg, Luxembourg; 2 Integrated BioBank of Luxembourg (IBBL), 1210 Luxembourg, Luxembourg; 3 Laboratory of Retrovirology, Public Research Centre for Health (CRP-Santé), 1526 Luxembourg, Luxembourg; 4 Life Sciences Research Unit, University of Luxembourg, 1511 Luxembourg, Luxembourg; 5 Laboratory of Plant Molecular Biology, Public Research Centre for Health (CRP-Santé), 1526 Luxembourg, Luxembourg; 6 Luxembourg Centre for Systems Biomedicine (LCSB), University of Luxembourg, 4362 Esch-sur-Alzette, Luxembourg

**Keywords:** Translational bioinformatics, Clinical bioinformatics, Translational research, Systems biology, Next-generation sequencing, Bioinformatic infrastructure

## Abstract

The 6^th ^Benelux Bioinformatics Conference (BBC11) held in Luxembourg on 12 and 13 December 2011 attracted around 200 participants, including internationally-renowned guest speakers and more than 100 peer-reviewed submissions from 3 continents. Researchers from the public and private sectors convened at BBC11 to discuss advances and challenges in a wide spectrum of application areas. A key theme of the conference was the contribution of bioinformatics to enable and accelerate translational and clinical research. The BBC11 stressed the need for stronger collaborating efforts across disciplines and institutions. The demonstration of the clinical relevance of systems approaches and of next-generation sequencing-based measurement technologies are among the existing opportunities for increasing impact in translational research. Translational bioinformatics will benefit from research models that strike a balance between the importance of protecting intellectual property and the need to openly access scientific and technological advances. The full conference proceedings are freely available at http://www.bbc11.lu.

## Background

In a time full of possibilities and expectations for personalised medicine, bioinformatics represents more than a technological convergence point at the intersection of various research fields.

Bioinformatics has become a bridge of integration. A continuous integration of information, insights and teams all over the planet. But it also catalyzes the translation of advances from the computing into the life sciences, and from there to the clinic, and back. Bioinformatics is gradually expanding its potential to improve the life of patients everywhere, through the transformation of fundamental discoveries into advanced approaches to preventing, detecting and treating disease [1,2].

We met at The 6^th ^BeNeLux Bioinformatics Conference (http://www.bbc11.lu, 12 and 13 December 2011, Luxembourg) to review advances, reflect on challenges and celebrate the contributions of bioinformatics in a "post-genome" era. The conference attracted around 200 participants, including internationally-renowned guest speakers and more than 100 peer-reviewed abstract submissions from 3 continents.

## Bioinformatics can answer "when", "what if" and "why" questions

Our programme of guest and selected oral and poster presentations comprised a diverse range of biologically and clinically relevant questions, computing approaches and applications [3]. These sessions were driven also by a significant interest in discussing challenges and directions for enhancing the impact of bioinformatics in fundamental and translational biomedical research. These features were specially reflected in the presentations given by our guest speakers: Burkhard Rost (Technical University Munich, Germany), Thomas Lengauer (Max-Planck-Institut Informatik, Germany), Ioannis Xenarios (Swiss Institute of Bioinformatics, Switzerland), and Peter van der Spek (Erasmus Medical Center, The Netherlands).

Presentations and discussions addressed applications of significant relevance to the detection and treatment of cancers, infectious, neurodegenerative and cardiovascular diseases. The meeting also showcased progress in bioinformatic infrastructures and enabling technologies. Examples of the latter included next-generation sequencing tools and applications, complex information integration and annotation over the Web, and several disease- and organism-specific research resources.

Among such a diversity of approaches and applications, a crucial pattern can be discerned. Bioinformatics research is putting a great emphasis on answering "when", "what if" and "why" questions using different types of biological data. When are complex events at the molecular and systems levels taking place ? When are specific disease states likely to occur? Thomas Lengauer's talk on translational bioinformatics in treating HIV infections represented an instance in this direction. What if specific perturbations are induced ? Why are observed associations or predictions biologically meaningful ? Ioannis Xenarios's talk on gene and cellular regulatory network models was an example within this research question category.

Different presentations also highlighted opportunities for integrating multiples vistas of biological phenomena as dynamic webs of interactions. Examples of this included: "Visualizing genotype-phenotype relationships across cell cycle and evolutionary time scales" (Maria Secrier and Reinhard Schneider), "Prediction of a phosphorylation network in Arabidopsis thaliana" (Kris Laukens et al.) and "Interfering with the interaction network of adenyl cyclase virulence factors" (Therese Malliavin et al.). This and future contributions would not be possible without advanced capabilities in large-scale data analysis and interpretation, for example from next-generation sequencing experiments (Alejandro Sifrim et al.; Tim de Meyer et al.) and from other types of "omic" data (Yan Wu et al.; Alexey Stukalov et al.).

Harnessing the huge amounts of data routinely produced by high-throughput techniques remains a challenge, as demonstrated by some of the posters presented during the conference. While microarrays still represent a fair amount of the contributions in genomics, next-generation sequencing has begun its journey towards clinical applications. This is happening thanks to the development of affordable, robust and well-standardized pipelines available for service, as introduced in "Union makes strength: building baseline Tracks from 69 open access full human genomes" (Stephane Plaisance and Mark Veugelers). Having all this information available, efforts are being put in its "organisation": building pathways, modelling networks, analysing interactions, and prioritising candidates for further downstream validation. "Analyzing gene and protein expression variance in cellular pathways using high-throughput experimental data" (Enrico Glaab and Reinhard Schneider) and "Network analysis of differential expression for drug target prioritization" (Griet Laenen et al.) are examples of work in this direction. Given all the methods proposed, it is also worthwhile to compare how they perform, as demonstrated in "Critical assessment of candidate gene prioritization methods" (Daniela Bîrnigen et al.).

Another remarkable point is how bioinformatics is expanding beyond research environments to reach the clinicians directly: "Breaching the surface with HOPE" (Jules Kerssemakers) aims at abstracting the complexity behind resources dealing with protein interfaces, surfaces and assemblies, thus helping physicians to understand the structural effects of mutations. Furthermore, one of the three awarded posters, "Clinical data miner - an electronic data capture software framework that improves interrater agreement" (Arnaud Installé et al.), was specifically aimed at the routine clinical environment. The proposed method enhances expert robustness and facilitates interpretation in situations where "case report forms" are used.

## Perspectives

Presentations and a panel-driven debate on "Technological and Cooperation Challenges in Bioinformatics" offered crucial insights into the state of our field, including critical research directions and advances to watch in the short and longer terms.

The need for cross-disciplinary cooperation in bioinformatics and in its different application domains is unassailable. If we want to develop new tools to improve human health in fundamental and clinical routine settings, new ways to share data and software will be essential. Many of such collaborations will ultimately be benefitted from different levels of data integration and access to high-performance computing. The selection of specific tools and approaches will continue being driven by domain-specific biological questions and computing requirements. For example, the use of cloud computing technologies cannot be seen as an "one-fits-all" solution in fundamental or translational biomedical research. The size and complexity of many projects requiring, for example, large-scale software development and integrative data mining demand a deeper involvement of area experts in key steps and decisions of the translational research cycle.

Researchers are incrementally breaking new ground to demonstrate the relevance of systems biology approaches to translational research with stronger couplings between *in silico*, *in vitro *and *in vivo *models. Although the estimation of the clinical relevance and utility of the majority of findings reported to date will require significant efforts, areas such as disease biomarker discovery and drug target prediction offer fertile ground for progress in the near future. The latter will be greatly facilitated by better access to carefully annotated sample cohorts (biobanking) and by the availability of user-friendly bioinformatic infrastructures.

The potential impact of bioinformatics in translational and clinical research will also depend on our capacity to harmonise the needs for freely publishing research outputs and for protecting intellectual property. This is particularly critical taking into account the advantages and opportunities offered by open-source development and open-access publishing.

Bioinformatics will be expected to contribute new ways to streamline the discovery pipeline needed for the development of diagnostics and drugs in the face of high costs and stringent regulatory standards. This will be possible through advances in the interrogation and simulation of complex dynamic systems, and based on the capacity of such models and resulting analyses to be reproduced and independently validated.

## Conclusions

The 6^th ^BeNeLux Bioinformatics Conference discussed advances and challenges in bioinformatics across a diverse range of applications. A key theme of the conference was the mission and roles of bioinformatics in enabling translational and clinical research. Although we did not intend to provide an in-depth discussion of opportunities and challenges in translational bioinformatics, a number of important perspectives were derived from the conference and deserved to be highlighted in this article. Major opportunities and challenges include the extraction of biological meaning from next-generation sequencing and other high-throughput "omic" technologies, the development of user-friendly bioinformatic infrastructure and the demonstration of the clinical relevance of systems-based approaches.

## Competing interests

The authors declare that they have no competing interests.

## Authors' contributions

FA drafted the manuscript with inputs or revisions from other authors. All the authors read and approved the manuscript.
